# Telmisartan Tablets Repackaged into Dose Administration Aids: Physicochemical Stability under Tropical Conditions

**DOI:** 10.3390/pharmaceutics14081667

**Published:** 2022-08-11

**Authors:** Anthony P. Ma, Sherryl G. Robertson, Beverley D. Glass

**Affiliations:** Pharmacy, College of Medicine and Dentistry, James Cook University, Townsville, QLD 4811, Australia

**Keywords:** telmisartan, stability, humidity, multicompartment compliance aid, repackaged, high performance liquid chromatography, adherence

## Abstract

Dose administration aids (DAAs) are commonly used to assist patients with chronic disease to manage multiple medications and thus improve adherence. Several brands of telmisartan, commonly prescribed for hypertension, are available in Australia. Manufacturer’s storage advice is to leave tablets in the blister strip until administered to patients. This study aimed to investigate the stability of telmisartan tablets when repackaged and stored in DAAs, to identify a brand, which is sufficiently stable to be repackaged. All available brands of telmisartan tablets in Australia, which contain different excipients, were repackaged into DAAs and stored at 30 °C, 75% RH for 28 days before screening, using visual inspection and physical testing. A candidate brand was then selected for physicochemical and photostability testing using pharmacopoeial methods. Repackaged Mizart^®^ tablets were shown to be sufficiently stable, when repackaged and stored under tropical conditions (30 °C, 75% RH) for 28 days. Several of the other brands were deemed inappropriate for repackaging, due to physical instability, highlighting the importance of considering not only the drug, but also excipients to ensure the stability of repackaged medicines. Although the repackaging of telmisartan tablets is not advised, this study provides evidence to support the Mizart^®^ brand as an option for pharmacists to recommend for repackaging.

## 1. Introduction

Dose administration aids (DAAs) are often recommended in the management of chronic disease, to improve the adherence to multiple medications and thus health outcomes for patients. The repackaging of medications into DAAs, involving their removal from original packaging, results in exposure to environmental conditions, which may adversely affect their stability [1]. The environmental conditions of elevated temperature and relative humidity (RH) can be problematic for medication storage in tropical areas [2]. DAAs provide varying levels of protection from humidity for repackaged medicines, due to lack of hermeticity [3,4], and in tropical areas there is potential for exposure to higher levels of temperature and humidity.

There is a lack of product-specific physicochemical stability data to guide pharmacists and other health professionals when repackaging medications [5]. The removal of medications from original packaging and repackaging into DAAs voids the original expiry dates provided by manufacturers [5]. Although available guidelines provide some general advice on the practice of repackaging medications, the lack of information on specific medications can limit pharmacists’ ability to make informed judgements [5,6].

Several studies have demonstrated the risks of both physical and chemical instability in environments of elevated temperature and humidity. Repackaged atenolol tablets stored at 40 °C, 75% RH for 28 days [7], sodium valproate immediate release (IR) tablets stored at 40 °C, 75% RH for 14 days [8], and warfarin tablets stored at 40 °C, 75% RH for 56 days [9] all failed dissolution tests. The increased dissolution time of a drug is directly associated with reduced bioavailability and may affect therapeutic efficacy [10]. Repackaged dabigatran capsules, stored at 30 °C, 75% RH for 28 days [11] and aspirin tablets, stored at 40 °C, 75% RH [12] failed chemical content tests, and were deemed unsuitable for repackaging into DAAs, due to the potential impact on therapeutic efficacy.

Varying stability has also been demonstrated for different brands of medications that contain the same active pharmaceutical ingredient (API). A study on different brands of repackaged atenolol tablets found that physicochemical response to storage conditions varied across brands of tablets, with the author [7] attributing this to differing formulations and coating, although no mention was made of specific excipients. Investigation into two brands of repackaged clozapine tablets also demonstrated varying API levels between different brands of tablets subjected to identical repackaging and storage conditions [13].

Although once considered inert, excipients are now recognized as impacting therapeutic efficacy [14] and product stability [15], as well as causing adverse reactions in patients [16] and incompatibilities with APIs [17]. Although excipients in compounded products have been studied less, it has been demonstrated for example that the lactose content of commercial tablets compromises the stability of isoniazid in compounded mixtures [18]. A field that remains unexplored is the role excipients play in the stability of repackaged medicines, particularly in humid environments, where moisture permeability of DAAs is generally unknown [19]. Since a wealth of knowledge on the hygroscopicity of excipients exists [20,21,22], these data might enable brand selection, where problems with physical stability have been identified in repackaged medicines, which may not be attributed to the API.

Telmisartan is an antihypertensive and represents 11.5% of the 213,568,055 prescriptions subsidized in the 2020–2021 financial year by the Pharmaceutical Benefits Scheme in Australia [23], with AUD 105.5 million of allocated government funding provided for DAA services for the same period [24]. The manufacturers of telmisartan tablets recommend that their products be stored below 30 °C (some below 25 °C), be protected from light and moisture, and that tablets should not be removed from their foil pack until required for administration [25]. The various brands of telmisartan tablets available in Australia contain different excipients (Table 1) in their formulations. There is currently no data to inform the safe repackaging of telmisartan tablets into DAAs, especially in hot and very humid (30 °C, 75% RH) tropical areas. This study aims to screen brands of telmisartan tablets available in Australia to identify a candidate brand of telmisartan for repackaging and physicochemical stability testing and to make recommendations to pharmacists about repackaging of telmisartan tablets.

## 2. Materials and Methods

### 2.1. Materials

Eight brands of telmisartan tablets (40 mg) were purchased from a local pharmacy: Micardis^®^ (Boehringer Ingelheim, Sydney, Australia), DRLA^®^ (Dr Reddy’s Laboratories, Melbourne, Australia), APO^®^ (Apotex, Sydney, Australia), Mizart^®^ (Alphapharm, Ipswich, Australia), GH^®^ (Generic Health, Melbourne, Australia), Teltartan^®^ (Arrow Pharma, Melbourne, Australia), Sandoz^®^ (Sandoz, Sydney, Australia), and Pharmacor^®^ (Pharmacor, Sydney, Australia). All tablets were tested within their original expiry periods, and where chemical analyses were performed, tablets with the same batch number were used. Webster-pak^®^ 28 (Webstercare, Sydney, Australia) medication packs (small, heat-sealed) were employed for tablet repackaging.

Telmisartan of 99.8% purity from Tokyo Chemical Industry (Tokyo, Japan) was used for high performance liquid chromatography (HPLC) standard. Methanol (Fisher, Geel, Belgium; HPLC Grade), sodium hydroxide (Ajax Finechem, Sydney, Australia; analytical reagent), sodium acetate (Ajax Finechem, Sydney, Australia; analytical reagent), glacial acetic acid (Ajax Finechem, Sydney Australia; analytical reagent), and potassium dihydrogen phosphate (Scharlau, Sentmenat, Spain; reagent grade) were used to prepare HPLC mobile phase and buffers.

### 2.2. Tablet Repackaging and Test Conditions

The World Health Organization classifies geographical areas into various climatic zones, with Zone IVa (30 °C, 65% RH) representing hot and humid tropical areas, and Zone IVb (30 °C, 75% RH) representing hot and very humid tropical areas [26]. Although northern Australia is officially classified into Zone IVa [26], the tropical coast experiences extremes of temperature (>30 °C) and relative humidity (>80%) during the summer months [27].

Each brand of telmisartan tablet was repackaged into individual compartments of Webster-pak DAAs before storage in a Binder (Tuttlingen, Germany) KBF720 climate chamber at 30 °C ± 2 °C and 75% ± 5% RH for 28 days. DAAs were removed from the climate chamber for visual inspection at days 7, 14, and 21. Control samples (day 0) consisted of tablets removed from their original packaging immediately prior to testing.

### 2.3. Screening of Telmisartan Brands

All brands of telmisartan tablets were subjected to visual inspection (at 0, 7, 14, 21, and 28 days) and physical tests (at 28 days) of weight uniformity, hardness, and thickness after repackaging and storage. Three brands were also tested for friability and disintegration to enable the final selection of the candidate brand.

#### 2.3.1. Visual Inspection

Visual inspection for tablets according to the International Pharmacopoeia [28] was performed on all brands of tablets at 0, 7, 14, 21, and 28 days of storage. All repackaged tablets (56) were inspected for damage, swelling, mottling, discolouration, any other visible changes, and adhesion to the DAA.

#### 2.3.2. Weight Uniformity, Thickness, and Hardness

Weight uniformity tests were performed on 20 tablets of each brand according to the uniformity of mass testing method, described in the BP [29]. Each individual tablet was weighed using a Sartorius (Goettingen, Germany) ENTRIS224I analytical balance.

The thickness of 10 individual tablets of each brand was measured using electronic digital calipers (no brand/manufacturer). Hardness testing was performed on 10 tablets of each brand according to the resistance to crushing of tablets method, described in the BP [29]. Each individual tablet was tested using a VanKel (Varian, Cary, USA) VK200 Tablet Hardness Tester, with tablets orientated with the score perpendicular to the plane of jaw movement.

#### 2.3.3. Friability and Disintegration

Friability and disintegration testing was performed on three selected brands of telmisartan tablets. Friability tests were carried out according to the friability for uncoated tablets testing method described in the BP [29] and a VanKel (Varian, Cary, USA) Friabilator. Disintegration testing was performed using the disintegration of uncoated tablets method, described in the BP [29]. Six tablets of each brand were tested using a VanKel (Varian, Cary, USA) VK100 automated disintegration tester, with water as the immersion fluid, maintained at 37.5 °C.

### 2.4. Physicochemical Stability Testing

Data from screening tests were analysed and, based on the results, the candidate brand of telmisartan tablets (Mizart^®^) was selected for physicochemical testing.

#### 2.4.1. Determination of Chemical Content

Telmisartan content was determined using high performance liquid chromatography (HPLC). The HPLC instrumentation consisted of Shimadzu (Kyoto, Japan) Nexera-i LC2040C system fitted with a Agilent (Middelburg, Netherlands) Pursuit XRs 5 µm C8 column (150 × 4.6 mm), operated at 40 °C. The mobile phase was a mixture of 70% methanol and 30% 10 mM sodium acetate buffer, adjusted to pH 5.0 with acetic acid. Injection volumes of 5 µL were used, with a mobile phase flow rate of 1 mL/min. The detector wavelength was set at 298 nm and the autosampler at a temperature of 15 °C. The HPLC method was validated (Table S1) for linearity, accuracy, and precision and specificity according to ICH guideline Q2 (R1) [30].

A calibration standard of telmisartan in 0.005 M sodium hydroxide in methanol was prepared. Dilutions ranging in concentration from 12–60 µg/mL (in 60% methanol and 40% 10 mM sodium acetate buffer, adjusted to pH 5.0 with acetic acid) were analysed and used to generate a standard curve. All samples were centrifuged (Eppendorf 5810R centrifuge, Hamburg, Germany) at 4000 rpm and 16 °C for 10 min immediately prior to analysis.

Tablet samples were analysed in accordance with the US Pharmacopoeia (USP) monograph for telmisartan tablets [31]. Twenty tablets were dissolved with sonication in 500 mL of 0.005 M NaOH in methanol. In triplicate, aliquots were centrifuged (Eppendorf 5810R centrifuge, 4000 rpm, 16 °C for 10 min) and the supernatant subsequently diluted with mobile phase. All samples and standards were centrifuged immediately prior to HPLC analysis.

#### 2.4.2. Photostability Testing

Photostability testing was performed on Mizart^®^ tablets repackaged into two Webster-pak DAAs. One was wrapped in aluminium foil and used to evaluate any thermal degradation of telmisartan during the test (dark control). The DAAs were placed in an Atlas (Linsengericht, Germany) Suntest XLS + fitted with a Solar ID65 filter and exposed to a minimum of 1.2 million lux hours of visible light (400–800 nm) and 500 wH/m^2^ of UV light (320–400 nm) as per the ICH guidelines Q1B [32]. The telmisartan content of the tablets was then determined using the validated HPLC method.

#### 2.4.3. Dissolution Testing

Dissolution testing was performed on Mizart^®^ tablets as per Test 1 described by the USP monograph for telmisartan tablets [31]. A VanKel (Varian, Cary, NC, USA) VK7000 paddle apparatus (Apparatus 2) [29] with a paddle speed of 75 rpm was employed. Phosphate buffer at pH 7.5 (900 mL) was used as the dissolution medium and maintained at 37.0 °C ± 0.5 °C. At timed intervals, 1.5 mL samples were withdrawn, centrifuged (Eppendorf miniSpin plus, Hamburg, Germany; 14,500 RPM, laboratory temperature for 10 min), and analysed for telmisartan content using the validated HPLC method.

### 2.5. Micardis^®^ Stress Test

To assess the chemical stability of telmisartan in tablets that physically degraded, twenty Micardis^®^ tablets were placed in glass beakers covered by petri dishes and stored at 30 °C ± 2 °C and 75% ± 5% RH for 28 days. After 28 days, each beaker was triple rinsed with methanol into a 500 mL volumetric flask and analysed for telmisartan content using the validated HPLC method. Micardis^®^ was chosen as tablets exhibited the most significant physical degradation of all brands during screening and physical testing.

### 2.6. Comparison of Moisture Absorption by Mizart^®^ and Micardis^®^ Tablets

Twenty tablets (Mizart^®^ and Micardis^®^) were individually weighed using a Sartorius (Goettingen, Germany) Cubis 125P five-figure balance, repackaged and stored at 30 °C ± 2 °C and 75% ± 5% RH for periods ranging from 1 to 14 days. Upon completion of each test, the individual tablets were subsequently reweighed, and the percentage weight gain calculated. The results were analysed using a two-tailed Mann–Whitney U-Test with *p* < 0.05, performed with SPSS version 27 (IBM, Armonk, NY, USA).

## 3. Results

### 3.1. Screening of Telmisartan Brands

Table 1 presents a comparison of the excipients present in the telmisartan tablet brands used in this study. After repackaging and storage at 30 °C/75% RH for 28 days, all brands exhibited surface changes, with blurring of tablet markings. The discolouration of Micardis^®^, Sandoz^®^, and Pharmacor^®^ tablets was observed after 7 days, which progressed to a pronounced change from white to yellow by day 28. The discolouration of APO^®^ tablets from white to cream was observed after 21 days, with the same colour change noted for Mizart^®^, GH^®^, and Teltartan^®^ tablets at day 28 (Figure S1).

The adhesion of tablets to the DAA surface was problematic for all brands except Mizart^®^. After 7 days at 30 °C/75% RH, 100% of Micardis^®^, 100% of Sandoz^®^, and >10% of DRLA^®^, tablets adhered to the DAA blister. This was less pronounced for GH^®^ and Teltartan^®^ with <10% of tablets for these brands adhering to the DAA surface at 28 days.

All tablet brands passed the BP requirement for weight uniformity [29] at day 0 and after 28 days of storage at 30 °C/75% RH. However, a weight increase was measured for all brands with Micardis^®^ showing the highest increase (11.5%) and DRLA^®^ the lowest (4.2%). Changes in tablet thickness for the brands after 28 days at 30 °C/75% RH was variable, ranging from −2.0% for Mizart^®^ and APO^®^ to +10.3% for Pharmacor^®^ (see Table S2 for all data from the physical tests).

Tablet hardness could not be measured for Micardis^®^ and Sandoz^®^ due to extensive softening after 28 days at 30 °C/75% RH. Of the remaining brands, Pharmacor^®^ (−31.4%) and DRLA^®^ (−16.2%) both decreased in tablet hardness while APO^®^ (64.4%), Mizart^®^ (56.1%), Teltartan^®^ (46.4%), and GH^®^ (44.7%) tablets all increased in hardness.

Friability and disintegration testing [29] was performed on Mizart^®^, GH^®^, and Teltartan^®^ tablets to further discriminate between these brands. After 28 days of storage at 30 °C/75% RH, all three passed the BP friability tests with <1.0% loss of mass. Similarly, all three brands had disintegration times of <15 min, thereby meeting the BP requirement for uncoated tablets [29]. An increase in disintegration time was noted for all brands in comparison with their respective day 0 results, in the order GH^®^ (7.2%), Mizart^®^ (13.3%), Teltartan^®^ (20.0%). However, of the three brands, Mizart^®^ had the shortest disintegration time at day 28 (8 min, 5 s).

### 3.2. Selection of Candidate Brand for Physicochemical Testing

Micardis^®^ and Sandoz^®^ tablets were excluded as candidate brands on account of the loss of tablet integrity and pronounced discolouration at day 28 (Figure S1, Table S2). The adhesion of tablets to the DAA was considered problematic and thus DRLA^®^, APO^®^, and Pharmacor^®^ were also excluded as >10% of tablets had adhered to the DAA by day 14. From the three remaining brands (Mizart^®^, GH^®^, Teltartan^®^), Mizart^®^ was selected as the candidate brand for physicochemical stability testing, due to the fastest disintegration time and a total lack of tablet adherence to the DAA at day 28.

### 3.3. Physicochemical Stability and Photostability Testing

Mizart^®^ tablets passed the USP telmisartan content requirement (≥90.0% to ≤110.0%) [31] after being repackaged and stored for 28 days at 30 °C/75% RH (Table 2). Similarly, tablets exposed to light according to ICH guidelines Q1B [32] also passed USP requirements, with minimal (<0.4%) drug loss observed due to thermal degradation, despite temperatures reaching 46 °C during photostability testing. Tablets exposed to light were, however, found to be discoloured, having changed colour from white to cream.

**Figure 1:**
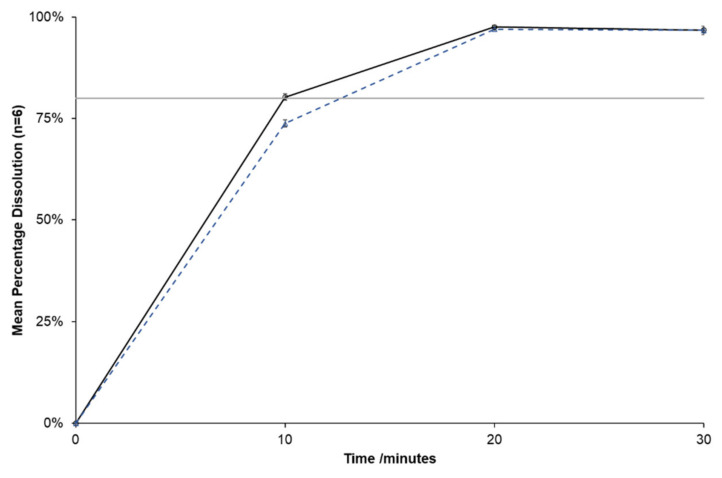
Dissolution profiles of Mizart^®^ tablets at day 0 (black) and after 28 days storage at 30 °C and 75% RH (blue, dotted). Data shown represent mean ± SEM for (n = 6) tablets.

Dissolution testing of Mizart^®^ tablets after repackaging and storage at 30 °C/75% RH for 28 days showed the brand passed the S_1_ level of testing with all six tablets exceeding 80% dissolution at 30 min (USP Dissolution Test 1 for Telmisartan Tablets) [31]. Comparing the dissolution profile for the repackaged tablets (28 days, 30 °C/75% RH) vs. those removed from the manufacturer’s packaging (day 0) showed the increased disintegration time impacted only the initial part of the dissolution curve (Figure 1).

### 3.4. Micardis^®^ Stress Test

Of the brands tested, Micardis^®^ displayed the poorest physical stability when repackaged, and, therefore, it was of interest to determine whether the physical degradation of the tablet had impacted the chemical stability of telmisartan. When exposed to 30 °C/75% RH in unsealed beakers, Micardis^®^ tablets were found to lose physical integrity and liquify by day 7. An analysis of the residue at 28 days however showed (Table 3) that the telmisartan content would have met USP requirements (≥90.0% to ≤110.0%) [31]. Although some degradation of telmisartan (4.92 ± 0.28%) was observed in the tablet residue, it is evident that the unsuitability of Micardis^®^ for repackaging and storage at 30 °C/75% RH is a consequence of physical instability.

### 3.5. Comparison of Moisture Absorption by Micardis^®^ and Mizart^®^ Tablets

**Figure 2:**
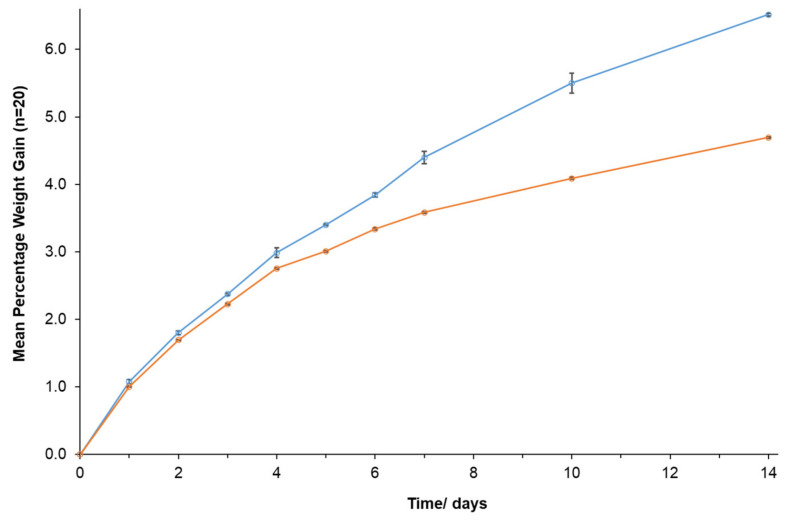
Comparison of weight gain for Micardis^®^ (blue) and Mizart^®^ (orange) tablets when repackaged and stored at 30 °C/75% RH. Data shown represent mean ± SEM for (n = 20) tablets.

The physical instability of telmisartan tablet brands when stored at 30 °C/75% RH was due to the ingress of water through the DAA. It was of interest to compare the relative rates of moisture absorption for the brand most affected (Micardis^®^) against the brand found to be physiochemically stable (Mizart^®^). Figure 2 shows the moisture absorption of repackaged tablets as measured by monitoring the weight gain of individual tablets for both brands. After 24 h storage at 30 °C/75% RH, the amount of moisture absorbed by Micardis^®^ was significantly different (*p* = 0.002) to that recorded for Mizart^®^. The surface of Micardis^®^ tablets were found to be pitted at day 3 and by the fourth day, the tablets were discoloured, sticky, and adhering to the DAA blisters. Measurements at days 10 and 14 required the Micardis^®^ tablets to be forcibly pulled from the blisters. In contrast, Mizart^®^ appeared unchanged except for slight discolouration at days 7 and 14. The physical dimensions of the tablets were similar (Table S3), the exception being the Mizart^®^ tablets having a greater thickness (4.34 ± 0.01 mm vs. 3.78 ± 0.01 mm for Micardis^®^).

## 4. Discussion

Telmisartan (Mizart^®^ brand) tablets were shown to be physically and chemically stable when repackaged and stored at tropical conditions of 30 °C and 75% RH for 28 days, meeting pharmacopoeial requirements [29,31]. Screening tests indicated that several other brands of telmisartan tablets were unsuitable for repackaging in tropical conditions, due to their physical instability. Although the removal of medications from their original packaging invalidates the manufacturer’s stability guarantee, repackaging in tropical areas introduces an additional degree of uncertainty around medication stability, due to high temperatures and humidity, especially due to the limited protection from humidity provided by the DAAs, due to their permeability [19]. Consequently, there is an increased risk of moisture uptake by medications, which may result in changes to the physical properties of a dosage form and/or the chemical instability of the API. For example, repackaged dabigatran capsules were considered chemically stable when stored at 25 °C (Canada, humidity not monitored) for 120 days [33]; however, when exposed to tropical conditions (30 °C/75% RH), the capsule contents degraded to a gel-like mass in 28 days and failed compendial drug content requirements [11]. Similarly repackaged aspirin tablets were chemically stable when stored at subtropical conditions of 25 °C/60% RH for 7 days, but exhibited a loss of potency at 40 °C/75% RH for the same time period [12]. In contrast, repackaged Mizart^®^ and Micardis^®^ tablets passed drug content requirements despite the latter losing physical integrity to the point of liquefying during the stress test.

All brands of telmisartan tablet exhibited some changes to their physical appearance during testing. Repackaged Mizart^®^ tablets discoloured from white to cream both during photostability testing and during storage in the climate chamber, with these changes to the physical appearance of tablets having the potential to impact patient adherence [12,34]. Tablet discolouration because of light exposure can, however, be addressed by the provision of a protective cardboard sleeve and patient counselling by pharmacists to store the DAA protected from light [35]. The protection of moisture-sensitive tablets from high humidity, especially given the limitation of the DAAs, cannot be addressed entirely by patient counselling, and this remains a challenge for pharmacists in tropical areas.

Medicines exposed to moisture can chemically degrade due to hydrolysis of the API, and/or physically degrade by moisture absorption of hygroscopic APIs or excipients. Identifying a patient-acceptable solution that enables repackaging first requires the chemical stability of the API to be determined under relevant conditions. If chemical stability is established and the API is not hygroscopic, a knowledge of excipient hygroscopicity will inform the selection of an alternative brand. Medicines containing APIs, which are susceptible to hydrolysis when repackaged, require either the complete exclusion of moisture, which is not possible when repackaging in a DAA, or a novel approach. The former may be achieved by repacking tablets/capsules in the manufacturer’s foil in the DAA; however, this may be problematic for patients, who might inadvertently try to take the tablet still wrapped in the foil or find it difficult to remove the foil [36]. The storage of DAAs in a domestic refrigerator has been demonstrated to enable repackaged dabigatran capsules [11] and sodium valproate EC tablets [37] to satisfy compendial requirements.

There has been limited investigation into brand-dependent stability of repackaged medications. The brand comparisons of repackaged atenolol [7], clozapine [13], and levothyroxine [38] tablets have been published; however, the studies failed to explore the impact of excipients on stability. In the current study, the choice of tablet filler (Table 1) was found to have a profound effect on the physical stability of telmisartan tablets. Brands containing sorbitol (Micardis^®^) and lactose (Sandoz^®^ and Pharmacor^®^) exhibited more prominent changes to tablet appearance, tablet weight, and hardness, when compared to brands containing mannitol (such as Mizart^®^, GH^®^, and Teltartan^®^). The hygroscopicity of sorbitol is known, and the substance has been shown to exhibit exponential weight gain at relative humidities >70% [22]. In contrast, mannitol has the lowest water uptake of the three fillers [22]. That a statistically significant difference in moisture absorption for Micards^®^ vs. Mizart^®^ tablets can be detected within 24 h highlights the potential advantage of mannitol as a tablet filler at conditions of high humidity.

It is, therefore, recommended that Mizart^®^ tablets are used when patients require telmisartan tablets to be repackaged into DAAs in tropical areas, with repackaging of other brands of telmisartan tablets to be avoided. Pharmacists should counsel patients about protecting DAAs from light and provide a means, such as a cardboard sleeve, to facilitate this protection. Consumer Medicine Information leaflets (CMIs) [25] can be useful for pharmacists to identify tablets that contain excipients, which may contribute towards tablet hygroscopicity, moisture absorption, and subsequent physical instability.

A limitation of this study is that the potential effect of excursions of environmental conditions above 30 °C and 75% RH in tropical areas was not measured. Some evidence supports the refrigerated storage of repackaged dabigatran capsules [11], aspirin tablets [12], levothyroxine tablets [38], and sodium valproate enteric coated (EC) tablets [37] to improve stability, but this practice did not suitably improve stability of sodium valproate IR tablets [8]. Further research is, therefore, required to assess the benefits and risks of storing DAAs in a refrigerator.

## 5. Conclusions

This study provides evidence to support pharmacists and health professionals in making informed decisions when repackaging telmisartan tablets in tropical areas, where high temperatures and humidity may increase the risk of medication instability, compared to more temperate locations. The screening of all available brands of telmisartan tablets found the physical stability of tablets was brand-dependent and several were deemed unsuitable for repackaging in tropical climates. The variable response of brands when exposed to elevated humidity and temperature indicates the active pharmaceutical ingredient, telmisartan, was not the primary cause of physical instability. Despite recommendations from manufacturers to avoid the removal of telmisartan tablets from original packaging, Mizart^®^ tablets passed compendial tests for photostability and physicochemical stability after repackaging and storage at tropical conditions (30 °C and 75% RH) for 28 days. Therefore, the repackaging of Mizart^®^ tablets provides an appropriate solution for pharmacists to recommend when all other options for a patient requiring a DAA have been exhausted.

## Figures and Tables

**Table 1:** Comparison of different excipients present in telmisartan tablet brands (NB: all brands also contained povidone, meglumine, sodium hydroxide, and magnesium stearate) [25].

Tablet Brand	Excipient
Micardis^®^	Sorbitol
DRLA^®^	Mannitol, Polysorbate 80
APO^®^	Mannitol, Sodium stearyl fumarate
Mizart^®^	Mannitol, Sodium stearyl fumarate
GH^®^	Mannitol, Sodium stearyl fumarate
Teltartan^®^	Mannitol, Sodium stearyl fumarate
Sandoz^®^	Lactose, Ludipress
Pharmacor^®^	Lactose, Crospovidone, Colloidal anhydrous silica

**Table 2:** Chemical content of Mizart^®^ tablets as percentage of labelled amount of telmisartan determined by HPLC.

Chemical Content Testing (30 °C/75% RH, Webster-Pak DAA)		Mizart^®^ Tablets
Percentage of labelled amount of telmisartan ± SEM ^†^	Day 0	99.53 ± 0.02%
	Day 28	99.67 ± 0.00%
	% Change	0.14 ± 0.02%
**Photostability testing (as per ICH guideline Q1B** **[32]** **, Webster-Pak DAA)**		**Mizart^®^ Tablets**
Percentage of labelled amount of telmisartan ± SEM ^†^	Dark control	100.43 ± 0.20%
	Light exposed	100.09 ± 0.24%
	% Difference	−0.34 ± 0.24%

^†^ Standard error of the mean for (n = 3) replicates.

**Table 3:** Chemical content of Micardis^®^ tablets as percentage of labelled amount of telmisartan determined by HPLC.

Stress Test (30 °C/75% RH, Open Beaker).		Micardis Tablets
Percentage of labelled amount of telmisartan ± SEM ^†^	Day 0	101.76 ± 0.13%
	Day 28	96.84 ± 0.25%
	% Change	−4.92 ± 0.28%

^†^ Standard error of the mean for (n = 3) replicates.

## Data Availability

The data presented in this study are contained in the article and Supplementary Material.

## Supplementary Materials

The following supporting information can be downloaded at: https://www.mdpi.com/article/10.3390/pharmaceutics14081667/s1, Figure S1: Visual comparison of telmisartan brands after repackaging and storage for 28 days at 30 °C/75% RH, Table S1: HPLC validation data, Table S2: Results from visual inspection and physical tests used to screen telmisartan tablet brands, Table S3: Physical dimensions of Micardis^®^ and Mizart^®^ tablets when removed from manufacturer’s packaging.
