# Supplementary material for: Telmisartan Tablets Repackaged into Dose Administration Aids: Physicochemical Stability under Tropical Conditions
Source: Pharmaceutics. 2022 Aug 11;14(8):1667. doi: 10.3390/pharmaceutics14081667 (PMC9414511; doi:10.3390/pharmaceutics14081667)
Supplement: Supplementary file 1 [file pharmaceutics-14-01667-s001.zip › pharmaceutics-1858582-supplementary.pdf]

Supplementary Material - Figures

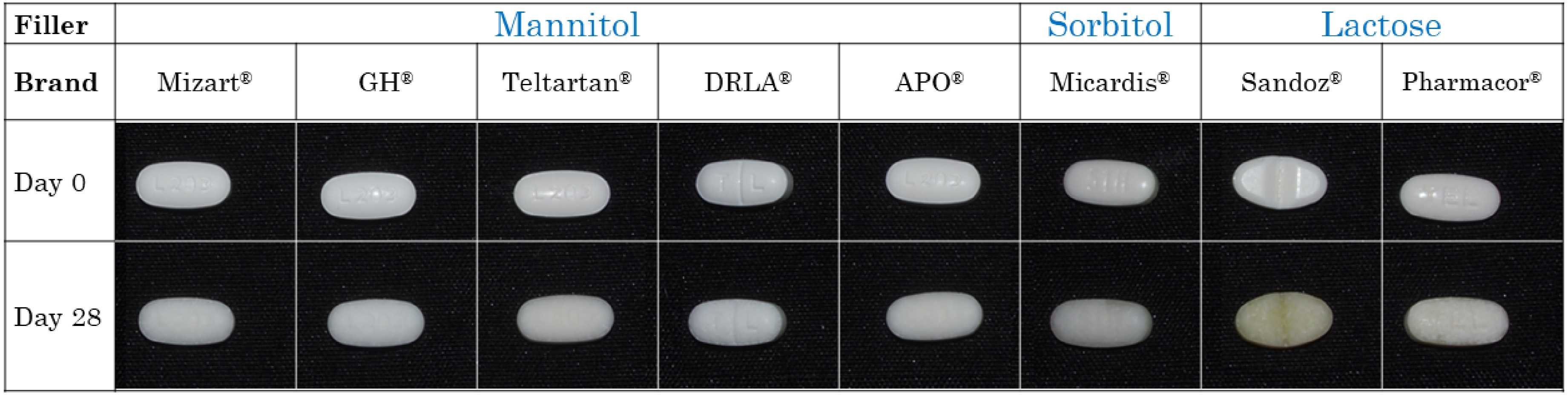

Figure S1. Visual comparison of telmisartan brands after repackaging and storage for 28 days at 30°C/75% RH.

Supplementary Material - Tables

Table S1. HPLC validation data

Telmisartan concentration (µg/mL)	% Relative Standard Deviation (%RSD)	% Relative Error (%RE)
12.02	0.30%	0.86%
36.06	0.18%	0.18%
60.10	0.48%	0.12%

Typical retention time	4.7 mins
Linearity (r^2)	> 0.99
Peak tailing factor (USP specification of NMT 2.0) [31]	1.4

Acidic conditions for forced degradation of telmisartan	0.5M hydrochloric acid, 50% acetonitrile, 60˚C	Comment: Specificity was demonstrated in the presence of tablet excipients and forced degradation products of telmisartan generated under acidic and alkaline conditions
Basic conditions for forced degradation of telmisartan	1M sodium hydroxide, 50% acetonitrile, 60°C	

Table S2. Results from visual inspection and physical tests used to screen telmisartan tablet brands.

**Tablet Brand**		**Mizart®**	**GH®**	**Teltartan®**	**DRLA®**	**APO®**	**Micardis®**	**Sandoz®**	**Pharmacor®**
Tablet filler		Mannitol	Mannitol	Mannitol	Mannitol	Mannitol	Sorbitol	Lactose	Lactose
**Visual Inspection**									
56 repackaged tablets were inspected for damage, swelling, mottling, discolouration, other visible changes, and fusion to the container.	Tablet intact	Yes	Yes	Yes	Yes	Yes	Yes	Yes	Yes
	Tablet discolouration	Yes, Day 28	Yes, Day 28	Yes, Day 28	No	Yes, Day 21	Yes, Day 7	Yes, Day 7	Yes, Day 7
	Tablet surface changes	Yes	Yes	Yes	Yes	Yes	Yes	Yes	Yes
	Tablets adhered to container<br>% of tablets adhered, day	No	Yes<br><10%, day 7	Yes<br><10%, day 14	Yes<br>>10%, day 7	Yes<br>>10%, day 7	Yes<br>100%, day 7	Yes<br>100%, day 7	Yes<br>>10%, day 14
**Physical Tests**									
**Uniformity of Weight**	Day 0 (mg)	240.3 ± 0.2	241.6 ± 0.4	241.4 ± 0.3	290.5 ± 0.3	240.4 ± 0.4	240 ± 0.4	238.8 ± 0.3	239.2 ± 0.9
Average weight ± SEM for 20 tablets	Test result	Pass	Pass	Pass	Pass	Pass	Pass	Pass	Pass
Test pass if no more than 2 tablets deviate by 7.5% from the average mass and no more than 1 tablet deviates by 15% from the average mass	Day 28 (mg)	253.1 ± 0.3	253.7 ± 0.3	254.6 ± 0.3	302.6 ± 0.4	254 ± 0.4	267.6 ± 1.7	258.8 ± 0.4	253 ± 0.8
	Test result	Pass	Pass	Pass	Pass	Pass	Pass	Pass	Pass
	% Average change	5.3%	5.0%	5.5%	4.2%	5.7%	11.5%	8.4%	5.8%
**Thickness**	Day 0 (mm)	4.34 ± 0.01	4.31 ± 0.01	4.25 ± 0.01	4.77 ± 0.01	4.31 ± 0.01	3.78 ± 0.01	3.43 ± 0	3.86 ± 0.01
Values are average thickness ± SEM for 10 tablets	Day 28 (mm)	4.25 ± 0.01	4.28 ± 0	4.18 ± 0.01	4.74 ± 0	4.23 ± 0.01	3.81 ± 0.02	3.53 ± 0.01	4.26 ± 0.02
	% Average change	-2.0%	-0.7%	-1.6%	-0.5%	-2.0%	0.7%	3.0%	10.3%
**Hardness**	Day 0 average (N)	75	79	87	164	71	133	126	129
Hardness for 10 tablets	Day 0 minimum (N)	61	70	79	149	56	124	116	115
	Day 0 maximum (N)	86	90	94	176	89	148	140	149
	Day 28 average (N)	116	115	127	137	117	Loss of tablet integrity	Loss of tablet integrity	89
	Day 28 minimum (N)	107	110	123	129	112			83
	Day 28 maximum (N)	123	120	130	180	125			93
	% Average change	56.1%	44.7%	46.4%	-16.2%	64.4%			-31.4%
**Friability**	Day 0 starting mass (28 tablets)	6.7064	6.7709	6.7733					
Each sample is whole tablets with a mass as close to 6.5g as possible	Day 0 finishing mass (28 tablets)	6.7085	6.7703	6.7565					
Test pass if loss of mass is not greater than 1.0%	Day 0 % mass change	0.0%	0.0%	-0.2%					
	Test result	Pass	Pass	Pass					
	Day 28 starting mass (26 tablets)	6.5776	6.5826	6.5766					
	Day 28 finishing mass (26 tablets)	6.5531	6.5760	6.5501					
	Day 28 % mass change	-0.4%	-0.1%	-0.4%					

	Test result	Pass	Pass	Pass
**Disintegration**	Day 0 (min:s)	7:08	8:20	7:31
Time of 6 tablets to disintegrate	Test result	Pass	Pass	Pass
Test pass if 6 tablets disintegrate within 15 minutes	Day 28 (min:s)	8:05	8:56	9:01
	Test result	Pass	Pass	Pass
	% Change	13.3%	7.2%	20.0%

Table S3. Physical dimensions of Micardis® and Mizart® tablets when removed from manufacturer's packaging.

	Micardis®	Mizart®
Shape	Oblong, convex	Oblong, convex
Length (mm)*	12.03 ± 0.01	12.08 ± 0.01
Width (mm)*	5.93 ± 0.02	5.93 ± 0.00
Thickness (mm)†	3.78 ± 0.01	4.34 ± 0.01
Weight (mg)†	240 ± 0.4	240.3 ± 0.2

* Mean ± SEM for (n=3) measurements.

† Mean ± SEM (n=20) measurements. Data replicated from Table S2.
